# Comparative Analysis of Full Genome Sequences of African Swine Fever Virus Isolates Taken from Wild Boars in Russia in 2019

**DOI:** 10.3390/pathogens10050521

**Published:** 2021-04-26

**Authors:** Ali Mazloum, Antoinette van Schalkwyk, Andrey Shotin, Alexey Igolkin, Ivan Shevchenko, Konstantin N. Gruzdev, Natalia Vlasova

**Affiliations:** 1 Reference Laboratory for African Swine Fever Virus, FGBI “Federal Centre for Animal Health” (FGBI “ARRIAH”), 600901 Vladimir, Russia; shotin@arriah.ru (A.S.); igolkin_as@arriah.ru (A.I.); shevchenko@arriah.ru (I.S.); gruzdev@arriah.ru (K.N.G.); vlasova_nn@arriah.ru (N.V.); 2 Agricultural Research Council—Onderstepoort Veterinary Institute, 100 Old Soutpan Road, Onderstepoort 0110, South Africa; VanSchalkwykA1@arc.agric.za

**Keywords:** African swine fever virus, Russian isolates, full-genome sequence, phylogenetic analysis, genome markers

## Abstract

In this study, we report on the full genome phylogenetic analysis of four ASFV isolates obtained from wild boars in Russia. These samples originated from two eastern and two western regions of Russia in 2019. Phylogenetic analysis indicated that the isolates were assigned to genotype II and grouped according to their geographical origins. The two eastern isolates shared 99.99% sequence identity with isolates from China, Poland, Belgium, and Moldova, whereas the western isolates had 99.98% sequence identity with isolates from Lithuania and the original Georgia 2007 isolate. Based on the full genome phylogenies, we identified three single locus targets, MGF-360-10L, MGF-505-9R, and I267L, that yielded the same resolving power as the full genomes. The ease of alignment and a high level of variation make these targets a suitable selection as additional molecular markers in future ASFV phylogenetic practices.

## 1. Introduction

African swine fever (ASF) is a hemorrhagic and frequently lethal disease of domestic pigs caused by African swine fever virus (ASFV). The etiological agent is a complex enveloped virus of icosahedral morphology with a double-stranded DNA genome [1]. The genome is divided into three main regions. The central region of about 125 kilo base pairs (kbp) is constant in length with less than 1.5% differences in size and is flanked by two highly variable regions at the ends [2,3]. Inside the central constant region there are zones of localized high variability, such as the CVR region within the B602L gene, produced by variations in the number of tandem repeats, some of which are used in order to discriminate between isolates [4].

African swine fever virus strains and isolates have been divided into 24 genotypes, based on the sequences difference of the C-terminal region of the B646L gene, which encodes the main variable capsid protein p72 [1,5,6,7]. Information based on the B646L gene alone is insufficient to track virus evolution across countries and regions, necessitating the use of sub-groupings based on the intergenic regions of the genome [4]. Another target for typing is based on the insertion of either one or two 10 base pair (bp) tandem repeat sequences within the intergenic region between the open reading frames (ORFs) I73R and I329L (IGR), subsequently dividing the isolates of genotype II into four sub-clusters [4,8,9]. Another 17-nucleotide tandem repeat sequence (TRS) insertion in the intergenic region between MGF-505-9R and MGF-505-10R (MGF) has been described to group isolates of genotype II into yet another three sub-clusters [10].

The disease is endemic in the majority of Sub-Saharan African countries and Sardinia, but in 2007, an outbreak was reported in Georgia that subsequently spread through the Trans-Caucasian region in 2007 to Europe (from 2014) and Asia (from 2018) [11,12]. Based on whole genome sequencing of the ASFVs isolated from these outbreaks, they belong to genotype II, with variants observed in the ORFs K145R, MGF-505-5R, and O174L, as well as in the IGR between I73R/I329L [9,13].

While control efforts, particularly increased biosecurity and professionalization of the pig industry, have successfully reduced the incidence of ASF in domestic pigs, the wild boar population represents a substantial reservoir in Europe and probably also in Asia, which will hinder eradication and serve as a source for further geographic expansion [9,14]. In the EU, over 30,000 cases in wild boar have been identified since 2014, and it can be assumed that the real number is much higher, since not all wild boar which succumb from the disease are found, tested, and reported [15]. Moreover, studies have shown that wild boars have their own epidemiological cycle including Eurasian wild boar (Sus scrofa), the wild boar habitat, and their carcasses [16]. In addition to the local transmission within the wild boar population, long distance jumps responsible for disease incursion into areas far from known infected regions occurred. In the EU, events of such long distance ASF spread have been described in the Czech Republic (Zlin area), Poland (area of Warsaw), Hungary, and Belgium [17]. These infected areas were each several hundreds of kilometers away from previously known infected regions.

According to OIE, 141 ASF outbreaks were reported in 2019 among wild boars (WB) (*n* = 62) and domestic pigs (DP) (*n* = 79) in the Russian Federation [18]. These outbreaks have two distinct geographic distributions either to the west or to the east of the country. The western outbreaks were widespread amongst 11 regions, spanning from Ulyanovsk and Nizhny Novgorod Oblast in the east, to the Black Sea and Caucasus in the southwest and Kaliningrad in the northwest. The outbreaks in the east were localized to three regions, Amurskaya, Primorsky Krai, and the Jewish Autonomous Oblast, along the border with the Peoples Republic of China. The wide distribution of ASFV in the Russian Federation and neighboring countries highlights the importance of full genome sequencing for understanding its global molecular epidemiology. The purpose of this study is to analyze the full genome sequence of four isolates taken from eastern and western regions of the RF in 2019 and compare them to isolates from neighboring countries.

## 2. Materials and Methods

### 2.1. Viral Isolates

**Figure 1:**
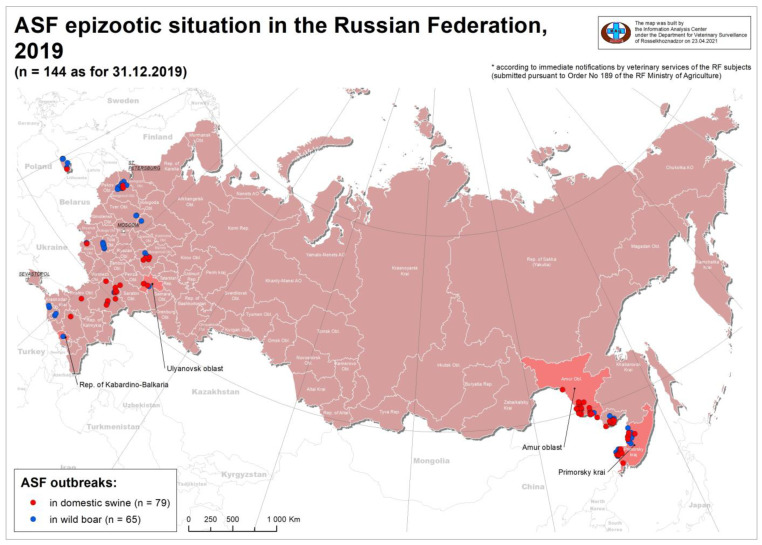
Map of African swine fever epizootic distribution in the Russian Federation in 2019. Red dots indicate the outbreak in domestic swine, while blue dots represents cases in wild boar. The Republic of Kabardino-Balkaria and Ulyanovsk Oblast in the west, and Amurskaya Oblast (Amur) and Primorsky Krai in the east of Russia are indicated on the map in dark pink.

Four samples (spleen) from dead wild boars (obtained from Primorsky Krai, Amurskaya Oblast, Ulyanovsk Oblast, and Kabardino-Balkaria Oblast) were sent to the reference laboratory for ASF at FGBI-ARRIAH for laboratory confirmation of the disease (Figure 1). Real time PCR (qPCR) was performed on the samples as described in the OIE manual using primers specific to the ASFV p72 gene [3,19,20]. The homogenates of PCR-positive tissue samples were used during virus isolation (VI) as described by Greig et al. in 1967 [21]. In short, the viruses were isolated using primary porcine spleen cell culture (PSC) in T-25 cell culture flasks containing cell suspension of 10 mL (in Eagle’s minimal essential medium—MEM containing 10% fetal bovine serum—FBS, penicillin 10^6^ U, gentamycin 0.008% and streptomycin 0.1 g/mL) [22]. The cell cultures were inoculated with 100 µL of 10% *w*/*v* filtered organ suspension and incubated at 37 °C for 7 days. Daily inspections of the cultures were performed using a light microscope in order to detect hemadsorption [22]. The titers of the ASFVs were determined by hemadsorption in PSC cell culture as described by de Leon et al. in 2013. Briefly, the PSC cells were added to a 96-well microtiter plate and inoculated with serial dilutions of 1:10 of the passaged ASFVs in octuplicate. Growth medium (MEM containing 10% fetal bovine serum—FBS, penicillin 10^6^ U, gentamycin 0.008% and streptomycin 0.1 g/mL) with 0.01% washed porcine homologous red blood cells (RBCs) were added to each well and incubated for 7 days at 37 °C and 5% CO_2_. The virus titer was calculated by the appearance of 50% of cases expressing hemadsorption as HADU_50_/cm^3^ [23].

The isolated viruses were passaged three times on PSC before harvesting and genomic viral DNA extraction. The virus isolates were designated as “ASFV/Primorsky 19/WB-6723”, “ASFV/Amur 19/WB-6905”, “ASFV/Ulyanovsk 19/WB-5699”, and “ASFV/Kabardino-Balkaria 19/WB-964”.

### 2.2. Statistical Analysis

Statistica (Statsoft, Tulsa, OK, USA) was used for statistical evaluation of the virus titer, and values were considered significantly different at *p* < 0.05. Virus titer was calculated using 96-well microtiter plate and inoculated with serial dilutions of 1:10 of the passaged ASFVs in octuplicate. Each titration was repeated 4 times (*n* = 4) for the calculation of standard deviation (SD) and *p* < 0.05.

### 2.3. Genome Extraction and Next Generation Sequencing (NGS)

Total genomic DNA (gDNA) of the ASFV isolates was extracted using the phenol-chloroform method as described by Szpara et al. in 2011 with some modifications [24]. Briefly, each virus was grown on PSC cell culture in six T-25 flasks for three days, when the cells were collected by centrifugation at 3000× *g* for 30 min at 4 °C following confirmation by hemabsorption. Each pellet was resuspended in cold, filtered PBS (Sigma, Merck, Darmstadt, Germany), and frozen at −70 °C, prior to repeated freezing and thawing at −70 °C and 37 °C in order to lyse the cells. The cellular debris was removed by centrifugation at 3000× *g* for 10 min at 4 °C, and the supernatant was moved to new tube. The supernatant was treated with 0.25 U/μL DNAseI (Evrogen, Moscow, Russian Federation) and 20 μg/mL RNAse A (Evrogen) and incubated for one hour at 37 °C, followed by the addition of protease from *Streptomyces griseus* (Sigma) to a final concentration of 10 U/mL and incubation at 37 °C o/n. The mixture was centrifuged on 30% sucrose at 15,890× *g* for 30 min at 4 °C and the pellet was resuspended in 300 μL PBS. Viral capsid was lysed by adding NaCl (final concentration of 1M), 200 μg/mL proteinase K (Sigma), and SDS (final concentration 1%) and incubated for 1 h at 56 °C, then extracted twice with phenol-chloroform. After centrifugation at 7000× *g* for 3 min, the upper layer was collected, and to 200 μL of the collected solution we added 10 μL 3M NaAc (pH 5.5) and 2 volumes of ice-cold 100% ethanol. The mixture was gently inverted and incubated at −70 °C for three hours, followed by centrifugation at 12,000× *g* for 30 min at 4 °C. The gDNA pellet was washed with 70% ethanol and resuspended in 50 μL TE buffer. The quality and quantity were measured using a spectrophotometer (Eppendorf, Merck, Darmstadt, Germany). A sequencing library was constructed for each of the samples using the Nextera XT DNA library preparation kit (Illumina, San Diego, CA, USA) and next generation sequencing (NGS) was performed using a MiSeq reagent kit version 2 with 2 × 250-bp paired-end sequencing on a MiSeq benchtop sequencer (Illumina, USA). Between 1,652,460 and 2,220,188 reads were obtained for each of the samples. The quality of the reads were determined and adaptors as well as low quality reads were removed using CLC Genomics Workbench v9 (Qiagen, www.clcbio.com). In order to assemble the genome, reads were mapped to the reference genome (FR682468.2_ASFV/Georgia 2007/1) using CLC Genomics Workbench v9. The trimmed reads were further mapped to their corresponding newly assembled virus genomes, with an average coverage depth of 45 times using CLC Genomics Workbench v9

Open reading frames (ORFs) were predicted using GATU software and the complete genome sequences were deposited in GenBank with accession numbers MT459800, MW306190, MW306191, and MW306192.

### 2.4. Phylogenetic and Single Nucleotide Polymorphism (SNP) Analysis

In order to perform the genetic analysis and comparison of isolates from the RF and neighboring countries, data of previously sequenced ASFV isolates were obtained from GenBank. These included sequences from China in 2018, Poland from 2017 to 2019, Belgium in 2018, Czech Republic in 2017, Moldova in 2017, Lithuania in 2014, Vietnam in 2019, inner Mongolia in 2019, Tanzania in 2017, and the revised sequence of the 2007 case in Georgia, ASFV/Georgia 2007/1 (FR682468.2). These sequences were used to generate an alignment, detect single nucleotide polymorphisms (SNPs), and determine the phylogenetic relatedness of the isolates. The alignment construction and SNP detection was performed using CLC Genomics Workbench v.9. 

Phylogenetic analysis of the sequences was performed by generating a maximum likelihood tree, with 1000 bootstrap iterations under the general time reversible (GTR, G + I = 4) model in Mega X [25,26]. 

Antigenicity plots of the predicted amino acid sequences were generated using the algorithm described by Welling et al. in 1985 [27]. This was performed using an 11-nucleotide sliding window in CLC Genomics Workbench V9. 

## 3. Results

Organs from dead wild boars were submitted from both the eastern and western regions of Russia in 2019 to FGBI-ARRIAH for laboratory confirmation of ASFV. Real time PCR positive samples were submitted for subsequent virus isolation. Porcine spleen cell (PSC) cultures were infected with organ suspensions, and hemabsorption was observed on the third day of incubation. These positive cell suspensions were consecutively passaged three times on PSC cell culture. The isolated virus titers were determined at each passage and an increase in virus yield was observed following every consecutive passage (*p* < 0.05) (Table 1). The increase in virus titer indicated that the virus replicates well in cell culture, with no apparent change to the hemabsorption activity through passages. 

**Figure 2:**
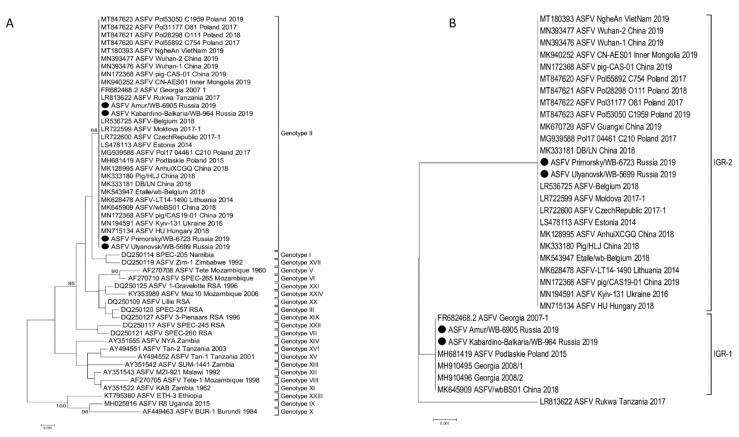
Maximum likelihood phylogenetic tree indicating the relationship of ASFV isolates based on their sequences of ORF B646L (**A**) and intergenic region IGR between I173R and I329R (**B**). The four isolates obtained in the Russian Federation are indicated with black circles.

**Figure 3:**
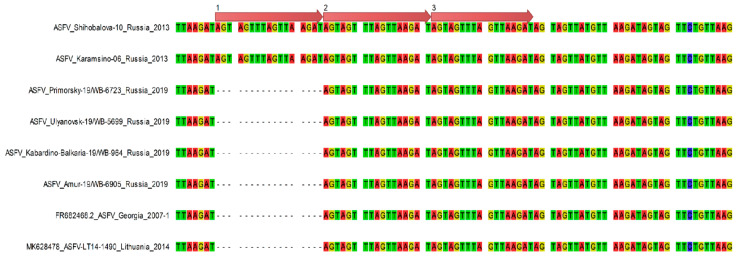
Nucleotide sequence alignment of the intergenic region between MGF-505-9R and MGF-505-10R genes of African swine fever virus strains belonging to B646L (p72) genotype II from the Russian Federation, Lithuania, and reference isolate Georgia 2007/1. Orange arrows on the top of the sequences indicates the number of repetitions of 17-nucleotide tandem repeat sequence (TRS) insertion.

**Figure 4:**
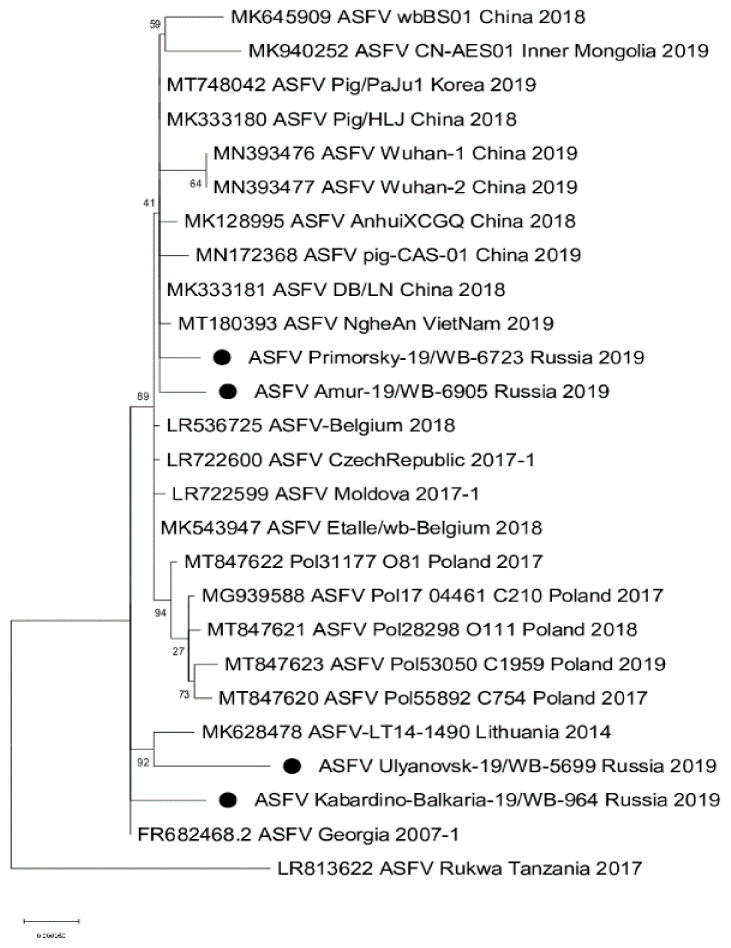
Maximum likelihood phylogenetic tree indicating the relationship of 16 complete ASFV genome sequences. The four isolates obtained in the Russian Federation are indicated with black circles.

Total genomic viral DNA was extracted from four isolates following the third passage on cell culture, the concentration of gDNA was around 7 ng/µL and a 260/280 ratio of ~1.8. These four isolates were referred to as ASFV/Primorsky 19/WB-6723, ASFV/Amur 19/WB-6905, ASFV/Ulyanovsk 19/WB-5699, and ASFV/Kabardino-Balkaria 19/WB-964, indicating the region where the samples originated. The complete genome sequence of each virus was determined using MiSeq NGS technology by mapping 163,115, 245,154, 198,319, and 164,563 reads, respectively to ASFV/Georgia 2007/1 (FR682468.1). The resulting complete genomes contained 189,263, 189,250, 189,398, and 189,384 nt, respectively, with 189 predicted protein-coding genes each. Phylogenetic analysis using the ~413 bp C-terminal region of ORF B646L, indicated that all four isolates belong to genotype II (Figure 2A). Analysis of the intergenic region between ORFs MGF-505 9R and 10R grouped them into subgroup MGF-1 (Figure 3; Table 2) [6]. In contrast, phylogenetic analysis of the 202 to 212 bp IGR sequences, between ORFs I73R and I329L, grouped isolates ASFV/Amur 19/WB-6905 and ASFV/Kabardino-Balkaria 19/WB-964 into IGR-1, whilst isolates ASFV/Primorsky 19/WB-6723 and ASFV/Ulyanovsk 19/WB-5699 were clustered into IGR-2 (Figure 2B). Phylogenetic comparisons using the complete genomes of the different ASFV isolates available on GenBank indicated a clustering of the eastern isolates ASFV/Primorsky 19/WB-6723 and ASFV/Amur 19/WB-6905 with the samples obtained from Europe and Asia (Figure 4). The isolates from the western regions of the RF, ASFV/Ulyanovsk 19/WB-5699 and ASFV/Kabardino-Balkaria 19/WB-964, cluster with the original Georgia/2007 (FR682468.2) isolate, a genotype II ASFV isolate from Tanzania (LR813622_Rukwa_Tanzania_2017), and the isolate from Lithuania in 2014 (MK628478) (Figure 4). 

**Figure 5:**
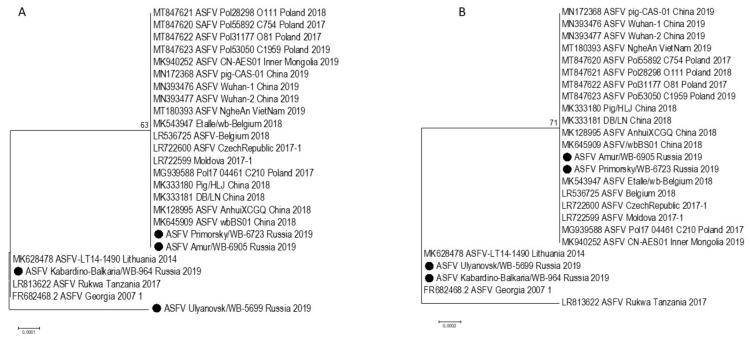
Subset maximum likelihood phylogenetic trees indicating the relationship of ASFV isolates based on their sequences of ORF MGF-505-9R (**A**) and ORF I267L (**B**). The four isolates obtained in the Russian Federation are indicated with black dots.

Single nucleotide polymorphisms (SNPs) were identified by using the previously generated alignment containing all the complete genome sequences. The nucleotide differences could be summarized as 13 single-nucleotide polymorphisms (SNPs) within the intergenic regions, 22 non-synonymous SNPs, 15 synonymous SNPs, and 7 SNPs altering the reading frame (Table 3 and Supplementary Table S1). The most significant of the 13 intergenic differences was the presence of the 10 bp internal repeat sequence (GGAATATATA) insertion between I73R and I329L [4], which separated the RF isolates ASFV/Ulyanovsk 19/WB-5699 and ASFV/Primorsky 19/WB-6723 with the insertion (IGR-2) from the two eastern isolates analyzed in this study (IGR-1) (Figure 2B). Seven of the intergenic changes were due to size difference in the homopolymer regions, while two of the SNPs were unique to ASFV/Primorsky 19/WB-6723, two SNPs unique to ASFV/Ulyanovsk 19/WB-5699, and one unique to ASFV/Kabardino-Balkaria 19/WB-964 (Supplementary Table S1). No differences in the intergenic region between MGF-505-9R and 10R were detected in the four Russian 2019 isolates, but non-synonymous SNPs were detected in MGF-505-9R, and the phylogenetic relation of genotype II ASFVs using the 1521 bp of this ORF was subsequently analyzed (Figure 5A).

Fifteen synonymous SNPs were identified in 14 proteins (Supplementary Table S1). The majority of the synonymous SNPs were either unique to ASFV/Ulyanovsk 19/WB-5699 (*n* = 6), or shared by ASFV/Amur 19/WB-6905 (*n* = 2) or Lithuania ASFV/LT14-1490/2014 (MK628478) (*n* = 2). In contrast, ASFV/Primorsky 19/WB-6723 had one unique synonymous SNP, while ASFV/Kabardino-Balkaria 19/WB-964 and ASFV/Amur 19/WB-6905 each had two unique SNPs (Supplementary Table S1). Open reading frame MGF-110-IL was predicted to terminate at three different sites. The first resulted in ASFV/Primorsky 19/WB-6723, ASFV/Amur 19/WB-6905, and all the European and Asian isolates terminating after 196 amino acids. The exceptions were Lithuania ASFV/LT14-1490/2014 (MK628478), Georgia/2007 (FR682468.2), and ASFV/Ulyanovsk 19/WB-5699, terminating after 214 amino acids, while ASFV/Kabardino-Balkaria 19/WB-964 and the African isolate Rukwa/Tanzania/2017 (LR813622) terminated after 269 amino acids.

The majority (*n* = 14) of the non-synonymous SNPs (*n* = 22) resulted in conservative amino acid exchanges (Table 3). The eight non-conservative amino acid exchanges were as follows: E323K and H456Y in ORF MGF-505-9R, K898N in ORF EP1242L, H129Y in ORF EP364R, E201K in ORF B602L, D276N in ORF B407L, Q104E in ORF E199L, and K90E in ORF I9R, highlighted in gray in Table 3. In order to determine if these ORFs containing SNPs could be utilized in future studies as molecular markers to distinguish between ASFVs belonging to genotype II, maximum likelihood trees of ORF MGF-505-9R (Figure 5A) and I267L (Figure 5B) were generated. Phylogenetic analysis using both these ORFs cluster ASFV/Ulyanovsk 19/WB-5699 and ASFV/Kabardino-Balkaria 19/WB-964 with the Georgia/2007 isolate, and ASFV/Amur 19/WB-6905 and ASFV/Primorsky 19/WB-6723 with the European and Asian isolates (Figure 5A,B). 

ASFV/Ulyanovsk 19/WB-5699 had the most unique non-synonymous SNPs (*n* = 7) and also had shared SNPs with ASFV/Kabardino-Balkaria 19/WB-964 and Lithuania ASFV/LT14-1490/2014 (MK628478) combined (*n* = 3) or with only the latter (*n* = 2). ASFV/Kabardino-Balkaria 19/WB-964 had five unique SNPs and shared one with ASFV/Primorsky 19/WB-6723. The latter had one unique SNP, but shared SNP with ASFV/Amur 19/WB-6905 and ASFV/AnhuiXCGQ/China/2018 (MK128995.1), supporting the clustering of isolates from the eastern region of the RF (Table 3). The possible impact of the non-synonymous SNPs on the function of the predicted proteins were investigated.

Gene MGF-505-9R contains two predicted non-conservative amino acid exchanges. The first is a lysine exchange of a glutamate at position 323 (E323K), which is located in ankyrin repeat motif of the predicted protein. Isolates ASFV/Ulyanovsk 19/WB-5699, ASFV/Kabardino-Balkaria 19/WB-964, the original Georgia/2007 (FR682468.2), and Rukwa/Tanzania/2017 (LR813622) have a positively charged lysine (K), in contrast to the acidic glutamate (E) at position 323. The second exchange H456Y has only ASFV/Ulyanovsk 19/WB-5699 exchanging the positive histidine (H) for polar tyrosine (Y) at position 456. 

Protein EP1242L is a predicted RNA polymerase subunit 2, with a hybrid-binding domain (new RNA/template DNA) between amino acids 813 and 989. Within this domain ASFV/Ulyanovsk 19/WB-5699 has a polar asparagine (N) at position 898 instead of the positive lysine (K) predicted for all the other ASFVs. Another nucleic acid binding protein, EP364R, has a predicted restriction endonuclease type II-like domain between amino acids 3 and 153, where ASFV/Amur 19/WB-6905 has a tyrosine (Y) at position 129 in place of a positive histidine (H). 

**Figure 6:**
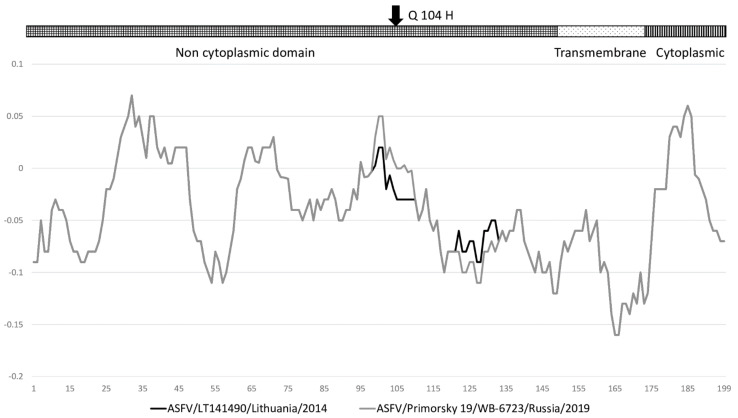
Antigenicity plots of predicted protein E199L of isolates Lithuania-LT141490/2014 (black) and ASFV/Primorsky 19/WB-6723 (gray), using the algorithm described by Welling et al. in 1985. A graphical representation of the predicted domains of E199L as well as the position of the amino acid exchange Q104H are indicated at the top of the graph.

Interestingly, both ASFV/Primorsky 19/WB-6723 and ASFV/Kabardino-Balkaria 19/WB-964 have different nucleotide substitutions, both resulting in a similar amino acid exchange of histidine (CA**T** or CA**C**), instead of glutamine (CA**A**) at position 104 of predicted protein E199L. E199L is associated with the inner virus envelope and predicted to play a role in virus entry [28]. The Q104H effective substitute is located within the non-cytoplasmic domain, and a determination of antigenicity using the algorithm described by Welling et al. in 1985 predicted that ASFV/Primorsky 19/WB-6723 and ASFV/Kabardino-Balkaria 19/WB-964 could have a positive antigenicity profile in comparison to other ASFVs (Figure 6). 

## 4. Discussion

As the number of ASF outbreaks in the territory of the RF and surrounding countries in Europe and Asia keeps increasing, it becomes harder for scientists to determine the spatial and temporal routes of ASFVs causing outbreaks, since all of the viruses belong to genotype II. Additional sub-clustering of ASF genotype II viruses could be performed by investigating a limited number of genomic targets: IGR of I73R-I329L and between MGF-505 9R-10R, and ORFs MGF-505-5R, C84L, O174L, MGF-110-9L, I267L, K145R, DP60R, and MGF-360-16R [4,8,9,10,29,30]. However, the current set of markers lack the phylogenetic resolution to draw justified conclusions when dealing with isolates from wild boars on the borders of two countries [31]. Therefore, additional complete genome sequences of new isolates should be performed and compared to previously published sequences in order to identify novel molecular markers and estimate genetic drift as well as the selection pressure enforced on the virus population.

Recently, it was shown that ASFV genotype II isolates could consist of a heterogeneous viral population, for example, Arm/07 [32], which could affect the sensitivity and analysis of full genome sequencing. Sequencing of heterogeneous populations usually requires additional clonal selection or plaque purification to obtain individual strains. The size and complexity of the ASFV genomes prohibits clonal selection, and plaque purification is time consuming. This could be mitigated by NGS and third generation sequencing (TGS) to evaluate changes in the complete or coding genome. ASF virus is prone to mutation as the features of viral X-type polymerase and DNA-ligase facilitate its genetic drift [33]. Infected domestic pigs cannot sustain a long-term replication cycle of the ASF virus due to the widespread implementation of stamping out policies or high mortality. Eradication of the viral host and the restriction of movement of infected products breaks the passing of possible mutated viruses. However, wild boars have their own epidemiological cycle, which includes the boars themselves, their habitat, and the consumption of carcasses [16]. Boars also have a high reproduction rate, therefore begetting a huge number of potentially susceptible descendants [34]. A sustained viral replication cycle allows for constant emerging and passaging of mutated viral variants within the same genotype. Moreover, there is evidence of viral mutations in wild boar populations occurring [35,36]. The original Georgian isolate was lethal to 100% of infected animals, but in 2014, Estonian wild boars have been tested seropositive, but negative for ASFV by PCR, implying that they have managed to survive the infection [37]. Similar results were also achieved in Poland in 2018 [30]. This illustrates that more resolving genome markers should be utilized for studying and analyzing the epidemiology of isolates, especially when full genome sequencing is not accessible.

Currently, an increasing number of full genome ASFVs sequences, isolated from countries around the RF, are published in Genbank. This study has contributed four additional full genome sequences of Russian ASF viruses isolated in 2019. Even though all the viruses belong to genotype II, they originated from two different regions in Russia, the east bordering China or from western regions. Full genome analysis of these four isolates establish geographical patterns, linking ASFV/Ulyanovsk 19/WB-5699 and ASFV/Kabardino-Balkaria 19/WB-964 with the Georgia/2007 isolate, and ASFV/Amur 19/WB-6905 and ASFV/Primorsky 19/WB-6723 with the European and Asian isolates (Figure 4; Table 2) [38].

The consensus genome sequences described in this study were generated by mapping 10% of the reads against Georgia-2007-1. The latter only contains a short (420 bp) region of the terminal inverted repeat region, and since the primary focus of this study pertained to detecting differences in the coding region of the genomes, attempts to resolve the terminal inverted repeats through K-mer selected de novo assemblies were not performed. The original reads were mapped to the newly generated consensus sequences in order to determine the average coverage of 45 times and identify low variant polymorphism based on frequencies with a cut-off value of 2% in order to account for sequencing errors. In order to identify additional genome markers, the complete genomes of the Russian isolates were compared to sequences available from GenBank. SNPs and indels were verified and reported if more than 98% of the reads contained the specific nucleotide change. Based on the identification of SNPs or indels within these genomes, ORFs MGF-360-10L, MGF-505-9R, and I267L were identified as possible genetic markers to discriminate between the closely related genotype II viruses. Phylogenetic analysis using these ORFs provided a discriminatory resolution similar to the same analysis with the full genomes. These ORFs are thus promising markers for resolving the phylogenetic relationships of ASFV isolates belonging to genotype II, and should be considered in future studies investigating the epidemiology of this disease. 

In addition to the phylogenetic resolution provided by full genome sequencing, it can provide valuable information concerning predicted amino acid exchanges (with non-synonymous being the most interesting) in proteins that play an important role in virus replication and virulence. Noteworthy examples of these amino acid exchanges are the K898N exchange in ORF EP1242L or E201K in ORF B602L of isolate ASFV/Ulyanovsk 19/WB-5699, the H129Y exchange in ORF EP364R of isolate ASFV/Amur 19/WB-6905, and the D276N exchange in ORF B407L of ASFV/Kabardino-Balkaria 19/WB-964. Identical exchanges of Q104H were detected in ORF E199L of both ASFV/Primorsky 19/WB-6723 and ASFV/Kabardino-Balkaria 19/WB-964, despite the usage of different codons to achieve this amino acid exchange. Additional analysis concerning the effect of these changes on virus replication and virulence is beyond the scope of this study, yet due to its possible importance, it should warrant additional investigation. The high number of amino acid exchanges described in ORFs predicted to encode nucleotide-binding enzymes was of interest (Table 3). These ORFs included EP1242L, a hybrid RNA/DNA binding RNA polymerase subunit 2; EP364R, which has a restriction endonuclease type II-like domain; O174L, the X-like DNA polymerase; NP419L, a DNA ligase; D1133L and Q706L, which both predicted that proteins belonged to the helicase super family II; and I243L, a predicted transcription factor SII (Table 3). This could indicate the possibility that the principle method of acquiring genetic diversity is not on the genetic level, but rather on the transcriptomic level [39]. Alternatively, it is known that the X-type polymerase and DNA-ligase in ASFVs have low fidelity and contribute to the increased genetic drift observed within this DNA virus [33,40]. The selective pressure enforced due to the changes in these nucleotide-binding enzymes were not calculated, but instead, their nucleotide polymorphisms were used in order to resolve the phylogenetic classification of these viruses. The Q104H effective substitution within the non-cytoplasmic domain of ORF E199L of isolates ASFV/Primorsky 19/WB-6723 and ASFV/Kabardino-Balkaria 19/WB-964 could have a positive antigenicity profile in comparison to other ASFVs (Figure 6). The functional influence of this exchange is challenging to predict, but is of interest since it occurred due to two independent homoplasious variations. The influence that each of these alterations in nucleotide binding enzymes have on the virus replication rate and efficiency should be investigated to determine their functional importance as possible targets for vaccine development, rather than their contribution as phylogenetic markers.

## Figures and Tables

**Table 1:** The calculated titer of individual ASFV isolates (*n* = 4) on cell culture, through three passage on PSC cells.

Virus Isolate	Virus Titer in Each Passage, lg HADU _50_/cm^3^ ± SD		
	1	2	3
ASFV/Primorsky 19/WB-6723	5.21 ± 0.36	6.66 ± 0.14	7.02 ± 0.12
ASFV/Amur 19/WB-6905	4.22 ± 0.21	5.10 ± 0.30	6.80 ± 0.45
ASFV/Ulyanovsk 19/WB-5699	5.10 ± 0.22	6.40 ± 0.14	7.21 ± 0.41
ASFV/Kabardino-Balkaria 19/WB-964	5.50 ± 0.36	6.20 ± 0.12	7.40 ± 0.14

**Table 2:** Molecular and spatial grouping of the Russian isolates described in this study.

Isolate	Origin	Genotype	MGF	IGR	Whole Genome
ASFV/Primorsky 19/WB-6723	Eastern	II	I	II	China 2018
ASFV/Amur 19/WB-6905	Eastern	II	I	I	China 2018
ASFV/Ulyanovsk 19/WB-5699	Western	II	I	II	Georgia/2007
ASFV/Kabardino-Balkaria 19/WB-964	Western	II	I	I	Georgia/2007

**Table 3:** Non-synonymous SNPs identified between the four Russian isolates, Georgia/2007-1 (FR682468.2), and AnhuiXCGQ/China/2018 (MK128995.1). The predicted proteins where the SNPs were identified, as well as the amino acid position, are indicated. The amino acid composition for each position within the aforementioned sequences are indicated with the polymorphism in bold. Non-conservative amino acid exchanges are highlighted in gray.

Protein	Position: Amino Acid	ASFV/Primorsky 19/WB-6723	ASFV/Ulyanovsk 19/WB-5699	ASFV/Amur 19/WB-6905	ASFV/Kabardino-Balkaria 19/WB-964	Georgia/2007-1 (FR682468.2)	ASFV/AnhuiXCGQ/China/2018 (MK128995.1)
MGF 110-9L	137: Y < C	C	C	C	**Y**	C	C
MGF 360-10L	329: S < N	**S**	N	**S**	N	N	**S**
MGF 505-4R	316: P < S	S	S	**P**	S	S	S
MGF 505-9R	323: K < E	E	**K**	E	**K**	**K**	E
MGF 505-9R	456: Y < H	H	**Y**	H	H	H	H
F317L	43: N < S	S	S	S	**N**	S	S
EP1242L	898: N < K	K	**N**	K	K	K	K
EP364R	129: Y < H	H	H	**Y**	H	H	H
B602L	201: K < E	E	**K**	E	E	E	E
B407L	276: N < D	D	D	D	**N**	D	D
CP2475L	1404: P < L	L	**P**	L	L	L	L
CP204L	141: V < A	**V**	A	A	A	A	A
O174L	168: H < R	R	**H**	R	R	R	R
NP419L	414: N < S	S	**N**	S	**N**	**N**	S
D1133L	457: S < P	P	**S**	P	P	P	P
H240R	186: H < R	R	**H**	R	R	R	R
Q706L	607: V < A	A	**V**	A	A	A	A
E423R	274: T < A	A	A	A	**T**	A	A
E199L	104: H = H < Q	**H**	Q	Q	**H**	Q	Q
I267L	195: I < F	F	**I**	F	**I**	**I**	F
I243L	44: F < Y	Y	Y	Y	**F**	Y	Y
I9R	90: E < K	K	**E**	K	K	K	K

Non-synonymous SNPs. Amino acid differences are indicated in **bold**. Amino acid exchanges with different charges are highlighted in gray.

## Data Availability

The data used to support the findings of the manuscript are included within the article.

## Supplementary Materials

The following are available online at https://www.mdpi.com/article/10.3390/pathogens10050521/s1, Table S1: Synonymous SNPs identified between the four Russian isolates, Georgia/2007-1 (FR682468.2) and AnhuiXCGQ/China/2018.
