# Supplementary material for: Comparative Analysis of Full Genome Sequences of African Swine Fever Virus Isolates Taken from Wild Boars in Russia in 2019
Source: Pathogens. 2021 Apr 26;10(5):521. doi: 10.3390/pathogens10050521 (PMC8146468; doi:10.3390/pathogens10050521)
Supplement: Supplementary file 1 [file pathogens-10-00521-s001.zip › pathogens-1178988-supplementary.pdf]

Supplementary Table S1. Synonymous SNPs identified between the four Russian isolates, Georgia/2007-1 (FR682468.2) and AnhuiXCGQ/China/2018. The predicted protein where the SNPs were identified as well as the amino acid position are indicated. The Nucleotide composition for each position within the aforementioned sequences are indicated with the polymorphism in bold.

Synonymous SNPs. Nucleotide differences are indicated in **bold** for six of the genomes.							
Protein	Position: Amino acid	ASFV/Primorsky 19/WB-6723	ASFV/Ulyanovsk 19/WB-5699	ASFV/Amur 19/WB-6905	ASFV/Kabardino-Balkaria 19/WB-964	Georgia/2007-1 (FR682468.2)	ASFV/AnhuiXCGQ/China/2018 (MK128995.1)
MGF 360-10L	134: C = C	A	**G**	A	A	A	A
MGF-360-11L	123: T = T	T	T	T	**C**	T	T
MGF 505-5R	299: K = K	G	**A**	G	G	G	G
MGF 505-10R	30: Y = Y	C	C	C	**T**	C	C
F1055L	84: N = N	A	**G**	A	A	A	A
F1055L	532: L = L	G	**A**	G	G	G	G
K205R	77 K = K	A	**G**	**G**	A	A	A
K20R	192: S = S	T	**G**	**G**	T	T	T
K421R	108: Q = Q	G	G	**A**	G	G	G
EP1242L	573: I = I	G	**A**	G	G	G	G
EP364R	68: A = A	G	G	**A**	G	G	G
B385R	98: L = L	C	**T**	C	C	C	C
CP2475L	1637: Q = Q	C	**T**	C	C	C	C
R298L	20: A = A	C	**T**	C	C	C	C
Q706L	606: C = C	**G**	A	A	A	A	A
SNPs within intragenic regions. Nucleotide differences are indicated in **bold** for six of the genomes.							

Intragenic region between ORF one	Intragenic region between ORF two	ASFV/Primorsky 19/WB-6723	ASFV/Ulyanovsk 19/WB-5699	ASFV/Amur 19/WB-6905	ASFV/Kabardino-Balkaria 19/WB-964	Georgia/2007-1 (FR682468.2)	ASFV/AnhuiXCGQ/China/2018 (MK128995.1)
MGF-360-3L	MGF-110-1L	**A**	G	G	G	G	G
MGF-360-3L	MGF-110-1L	**-**	**T**	-	**T**	**T**	-
ASFV-Ch-ACD-00290	ASFV-Ch-ACD-00300	**--**	**GG**	**GG**	--	--	--
MGF-300-1L	MGF-300-2R	**GG**	**GG**	**GG**	**GG**	--	G-
MGF-300-1L	MGF-300-2R	A	-	A	A	A	A
MGF-360-11L	MGF-505-1R	**A**	G	G	G	G	G
C62L	C962R	T	**C**	T	T	T	T
B602L	B385R	G	-	G	G	G	G
CP123L	CP2475	-	-	-	**T**	-	-
I73R	I329L	+ 10bp	+ 10bp	-	-	-	+ 10bp
I329L	I215L	G	G	G	**A**	G	G
